# Plasma Amino Acid Concentrations After the Ingestion of Dairy and Collagen Proteins, in Healthy Active Males

**DOI:** 10.3389/fnut.2019.00163

**Published:** 2019-10-15

**Authors:** Rebekah D. Alcock, Gregory C. Shaw, Nicolin Tee, Louise M. Burke

**Affiliations:** ^1^Mary Mackillop Institute for Health Research, Australian Catholic University, Melbourne, VIC, Australia; ^2^Australian Institute of Sport, Australian Sports Commission, Canberra, ACT, Australia; ^3^High Performance Unit, Swimming Australia, Brisbane, QLD, Australia

**Keywords:** leucine, proline, glycine, hydroxyproline, tendon, ligament, connective tissue, athlete

## Abstract

**Introduction:** Recent evidence suggests that the consumption of essential amino acids (AA) and/or those abundantly present in collagen may have the capacity to influence the synthesis of new collagen in ligaments and tendons, when tissue perfusion is optimized (e.g., during exercise). However, little is currently known about the bioavailability of these AAs in blood after the consumption of various collagen and diary protein sources: such information is needed to develop potentially useful dietary and supplement intake strategies.

**Objectives:** The aim of the current study was to characterize blood AA concentrations in response to consumption of collagen and dairy protein sources; specifically, maximum concentrations, the timing of maximum concentration, and total (area under the curve) exposure above baseline.

**Methods:** A 20 g serve of various dairy and collagen proteins, and a 300 mL serve of bone broth were consumed by healthy, recreationally active males after an overnight fast. Blood samples were drawn every 20 min for a total of 180 min, for analysis of plasma AA concentrations. Total AA, essential AA and collagen specific AAs were analyzed for maximum concentration, timing of peak, and area under the curve.

**Results:** In general, protein intake was associated with a similar increase in total and collagen specific AAs, except for collagen proteins being a superior source of glycine (683 ± 166 μmol/L) compared to 260 ± 65 μmol/L for dairy proteins (*P* < 0.0001), whilst dairy proteins were a superior source of leucine (267 ± 77 μmol/L) compared to 189 ± μmol/L for collagen proteins (*P* < 0.04). Although there were several differences in the bioavailability of hydrolysed compared to non-hydrolysed proteins, this only reached statistical significance within the dairy proteins, but not for collagen proteins.

**Conclusions:** The intake of collagen proteins result in higher plasma peaks of glycine, whilst the intake of dairy proteins result in higher plasma peaks of leucine. This information may support further investigations, and identification of key AAs that may support exercise in the synthesis of collagen.

## Introduction

Literature suggests that ligaments and tendons are similar to muscle in being dynamic structures that respond to mechanical loading with tissue hypertrophy (1–3). Larger tissues with more densely packed collagen fibrils have a greater capacity to withstand force, and thereby exert greater injury protection (4). On the other hand, smaller tissues with disorganized collagen fibrils have been associated with higher injury risk, such as the development of tendinopathies (1). Although the amino acids (AAs) required for myofibrillar tissue formation have been well-documented (5–9), evidence regarding the potential role of collagen precursors (e.g., proline, glycine and lysine) and/or stimulatory AAs (e.g., leucine) in the synthesis of new collagen in ligaments and tendons is still emerging. Furthermore, the requirement for an appropriate exercise stimulus to support tissue perfusion appears essential (10–13).

Leucine, an essential amino acid (EAA), has been the subject of extensive research for its role in stimulating myofibrillar protein synthesis (6). Leucine exerts its effect on the mammalian target of rapamycin (mTOR), which results in a signaling cascade leading to the synthesis of myofibrillar protein in muscle tissue (14). In the context of connective tissue, work undertaken in an animal model has suggested an increase in collagen synthesis in the deep digital flexor tendon of malnourished rats in association with a leucine-rich diet, and further enhanced when combined with physical stimulation (15). Similarly, in humans, the ingestion of leucine-rich whey protein, coupled with resistance training, has been shown to lead to patellar tendon hypertrophy, with the proximal cross-sectional area (CSA) increasing by 14.9 ± 3.1%, compared to 8.1 ± 3.2% for the placebo group (16), but no change in distal or mid tendon CSA. However, it is highly plausible that this increase occurred secondary to a distinct increase in the size of the quadriceps muscle, acting as a stimulatory load on the tendon (17).

In addition to the stimulatory role of leucine on the protein synthetic machinery, it is likely that other AAs, such as those found in large quantities in collagen protein (e.g., glycine, proline, and lysine) play a role in the synthesis of new collagen. Proline is a conditionally essential amino acid (CEAA), which plays a role in the formation and structural integrity of collagen fibrils (18). Indeed, in older individuals, ingestion of leucine and proline in the form of casein protein, in combination with resistance training, resulted in a trend toward a higher fractional synthetic rate of collagen compared to whey protein (19). In an engineered ligament model, it has been shown that the addition of 50 μM proline with 50 μM ascorbic acid, to a media rich in AAs such as leucine and glycine, increased ligament collagen content from 1.34 ± 0.2% to 8.34 ± 0.37% (20). More recently, work from our group has shown that the consumption of 15 g of dietary collagen; 1 h prior to intermittent exercise led to an increase in procollagen I N-terminal propeptide (P1NP), compared to a placebo control (21). Additionally, an *in vitro* arm of the same study, displayed an increase in the collagen content and mechanical properties of an engineered ligament treated with serum obtained post ingestion of dietary collagen (21). Thus, it is plausible that a combination of EAA, to upregulate synthetic machinery, and CEAA to supply AA building blocks is required during the synthesis of collagen protein. Although these findings are promising, it remains unclear as to the value of hyper-aminoacidemia in the synthesis of new collagen, and whether these amino acids work in isolation or synergistically.

In line with this, there is emerging evidence of benefits associated with the ingestion of collagen peptides in a range of collagen containing tissues, including increased collagen synthesis (22, 23), improved body composition (24, 25), reduced pain (26–28) and the slowing of degenerative diseases such as osteoarthritis (29, 30). It has been suggested that hydrolysis of collagen protein prior to ingestion allows two and three amino acid peptides to pass across the mucosal barrier equating to a higher expression and therefore biosynthesis within the tissue matrix (31). This was illustrated in a recent study whereby the consumption of hydrolysed collagen proteins resulted in a higher bioavailability of AAs compared to non-hydrolysed collagen protein and a placebo control (32). Furthermore, it has recently been shown that the consumption of collagen peptides resulted in a higher expression of collagen signaling proteins, compared to a placebo control (24).

Accordingly, we aimed to determine the bioavailability [i.e., timing, maximum concentration, and area under the curve (AUC)] of TAA, EAA, and key AAs proposed to support the synthesis of new collagen, after the consumption of a selection of hydrolysed and non-hydrolysed collagenous, and dairy proteins, and a collagenous food source. This data would be of benefit to allow further investigations into a range of practical questions:

- The optimal food source/supplement, AA and/or combinations of AAs that may support exercise in increasing collagen synthesis.
- The optimal timing to consume this food source/ supplement and/or AA(s) prior to an exercise bout.
- Whether there is increased bioavailability from the consumption of hydrolysed vs. non-hydrolysed forms of collagenous and/or dairy proteins.

## Materials and Methods

### Subjects and Ethics

Fifteen healthy, recreationally active, male subjects (30 ± 5 years; 80 ± 8 kg BM) with no current collagen-related disease or known protein allergies were recruited for the study. The current study formed part of a larger study, which necessitated a male subject population given the influence of female sex hormones of ligament health (33). Sample size was chosen using power estimation determined in previous, similar studies (34, 35). The Human Ethics Committee of the Australian Institute of Sport granted approval for this study (20170607) and written informed consent was received from participants prior to its commencement. The protocol was registered with the Australian New Zealand Clinical Trials Registry (ANZCTR12617000923369).

### Protein Source and Preparation

Four collagen and two high leucine dairy protein sources were selected for the study. These included a hydrolysed and non-hydrolysed collagen powder: Gelita (Pep) and gelatin (Gel), respectively; a hydrolysed and non-hydrolysed dairy protein supplement: calcium caseinate (Cas) and hydrolysed casein (HCas), respectively; a liquid collagen supplement (LCol) and one collagenous food source: bone broth (BBr). Further details of these protein sources can be seen in Table 1. We chose a standard dose of 20 g for all powdered supplements and 60 mL of liquid collagen (equivalent to 20 g of collagen protein as per manufacturer information). This dose was slightly higher than used in previous work (21), due to the benefits associated with increased amino acid availability (36, 37). The bone broth used in this study was chosen on the basis of the results of our previous work (38) in which this preparation was found to have a higher protein content than other broths assessed, and within the range provided by the reference supplements in a standard serve [further details of the broth used in this study; “chef prepared bone broth” are available elsewhere; (38)]. Furthermore, we decided on a 300 mL serve as a representative portion size that would be practical to consume within the prescribed time-period, as we would not ascertain total protein content of this broth prior to commencing the study. Analysis of the batch actually consumed in this study, subsequent to the trials, found that it was considerably higher than expected. Nevertheless, by purchasing a bulk volume of the broth (5 L), and mixing it well before dividing it into 300 mL serves, we achieved consistency between the serves consumed by subjects.

**Table 1:** Details of collagen and non-collagen (dairy) protein sources, and collagen specific amino acid profiles per serve of select food/supplement.

**Type**	**Dairy protein supplement**		**Collagen protein supplement**			**Collagen protein food source**
Brand	Professional whey, NSW, Australia	Professional whey, NSW, Australia	McKenzies, Altona, VIC, Australia	Tendeforte, Gelita, Eberbach, Germany	GBR Nutrition, Lancashire, UK	Elemental Cafe, Braddon, ACT, Australia
Details	Calcium caseinate	Hydrolysed casein powder	Powdered gelatin	Hydrolysed collagen peptide powder	Liquid collagen supplement	Chef made bone broth
Abbreviation	Cas	HCas	Gel	Pep	LCol	BBr
Serve	20 g	20 g	20 g	20 g	60 mL	300 mL
Cost per serve	$ 0.70	$ 1.24	$ 0.74	$ 3.00	$ 5.37	$ 4.50
Manufacturer stated total protein per serve (g)	20	20	20	20	20	N/A
TAA per serve (mg)^*^	19,172	19,378	20,108	20,594	17,892	52,260
EAA per serve (mg)^*^	8,306	8,368	4,944	5,858	4,392	13,320
Proline per serve (mg)^*^	2,038	2,060	2664	2,722	2,406	6,420
Glycine per serve (mg)^*^	360	354	4,750	4,904	4,212	11,760
Lysine per serve (mg)^*^	1,422	1,468	696	746	630	1,740
Leucine per serve (mg)^*^	1,782	1,786	588	584	516	1,710

*EAA, Essential Amino Acids; TAA, Total Amino Acids*.

* *Calculation based on free amino acid molecular weight*.

*Cysteine and typtophan have not been included in analysis, which account for <5% in total*.

To maintain consistency across all experimental protocols, all protein sources were served warm, and in/with the same amount of fluid (300 mL) achieved via the addition of water. Supplements were provided in a counter-balanced, randomized fashion, with at least 48 h between trials. All protein sources were assessed for full amino acid profiles analyzed using Ultra Performance Liquid Chromatography (Waters AccQTag Ultra) (Australian Proteome Analysis Factory, Macquarie University, Sydney, NSW).

### Experimental Protocol and Analysis

Participants fasted overnight (>10 h) prior to attending the laboratory at the Australian Institute of Sport between 5:30 and 7 a.m. On waking, participants were allowed to consume 250 mL of water to ensure adequate hydration for the blood collections but instructed to arrive to the lab in a rested state to avoid significant elevations to heart rate that would increase blood flow. On arrival, a 22 G indwelling cannula was inserted into the antecubital vein for blood collection by a trained phlebotomist, and a baseline (BL) blood sample was collected. Immediately after, the protein source was prepared and given to participants who were instructed to consume it within 5 min, sipping slowly throughout. Completion of the 5 min period was considered as *t* = 0. To standardize gastric emptying, no other fluid was available for 60 min following consumption, and then *ad libitum* water consumption was permitted. Immediately after the consumption of the protein source, participants were asked to fill out a feedback form (as outlined below). Blood samples were then collected every 20 min for 180 min into 2 mL lithium heparin Vacuette tubes (Greiner Bio-One, Kremsmünster, Austria). Immediately after each blood collection, samples were centrifuged at 1,500 × g for 10 min at 4°C. The resulting plasma was separated and stored at −80°C until further analysis. Once the 180 min period was concluded the cannula was removed and participants were allowed to leave the laboratory.

Once all plasma samples were collected, they were transported to Maastricht University for analysis of full amino acid profiles using liquid chromatograph mass spectrometry (UPLC-MS; ACQUITY UPLC H-Class with QDa; Waters, Saint-Quentin, France). Briefly, samples were prepared according to manufacturer's instructions, with an internal standard added. Fifty microliters of blood plasma was deproteinised using 100 μL of 10% sulfosalicyclic acid (SSA) with 50 μM of metabolomics amino acid mix standard (MSK A2) (Cambridge Isotope Laboratories, Massachusetts, USA). Subsequently, 50 μL of ultra-pure demineralized water was added and samples were centrifuged. After centrifugation, 10 μL of supernatant was added to 70 μL of Borate reaction buffer (Waters, Saint-Quentin, France). Twenty μL of AccQ-Tag derivatising reagent solution (Waters, Saint-Quentin, France) was then added, and the solution was heated to 55°C for 10 min. Of this 100 μL derivative, 1 μL was injected and measured using UPLC-MS.

Due to the interests of this study, we have reported concentrations of TAA, EAA, and a selection of AAs which have been suggested to be involved in the synthesis of collagen (including proline, glycine, lysine and leucine) (16, 19, 21, 39).

### Protein Source Acceptability

The feedback form completed by subjects immediately after consuming a test product provided four questions in the format of a Likert scale (40), and one question with a yes or no response. The questions are outlined in **Table 3** and included questions related to acceptability and palatability. While we acknowledge that this is not a validated questionnaire, it was intended that this feedback would provide practical insight into the use of this product for connective tissue health purposes.

### Statistics

Trapezoidal rule (41) adjusted to baseline concentration was applied to calculate the area under curve (AUC) of the amino acid concentration. Statistical analysis were performed using GraphPad Prism version 7.00 for Windows (GraphPad Software, La Jolla California, USA). Data were checked for normality using a D'Agostino & Pearsons normality test. A repeated-measures One-way ANOVA by General Linear Models (GLM) was used to compare the effects of different supplements on outcome variables (plasma amino acid content for: baseline; BL, observed maximum concentration; C_max_ Obs, timing of peak; T_max_ and AUC). Tukey's method was used to adjust for multiple comparisons between different groups.

## Results

### Total Protein and Amino Acid Content of Selected Collagen and Dairy Proteins

The composition per serve of the protein sources (total protein, TAA, EAA and selected AAs) is displayed in Table 1. Full amino acid profiles are available as Supplementary Table 1. The supplements (Cas, HCas, Gel, Pep, and LCol) provided 19.3 ± 0.8 g protein per 20 gram serve, whereas BBr provided 52.2 g of protein per 300 mL serve. Per serve, BBr contained the highest levels of all selected AAs, with the exception of leucine, which was provided in the highest amount in the dairy protein supplements, HCas and Cas at ~1,540 mg per serve. AA profiles of proline, glycine, lysine and leucine of protein sources of similar derivatives (e.g., dairy and collagen proteins), were comparable in their amino acid content. Liquid collagen provided the lowest levels of all AA among the collagen protein sources in this study.

#### Observed Maximum Concentration (C_max_ Obs)

**Figure 1:**
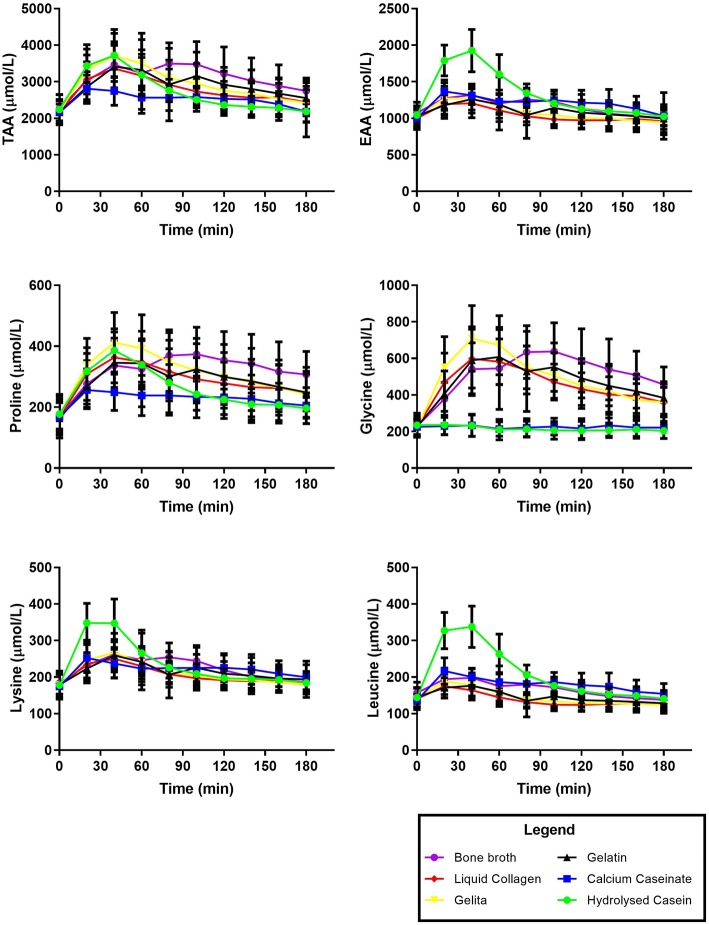
Time course of plasma amino acid concentrations over 180 min after the consumption of collagen or dairy protein sources. Data presented as mean ± SD.

Figure 1 displays the time course of the appearance of TAA, EAA, and the selected AAs in the plasma over 180 min. Kinetic parameters of TAA, EAA, and selected AAs after the consumption of collagen and dairy protein sources are displayed in Table 2, as means and SD. There were no differences in BL levels for TAA, EAA or any of the AAs between protein sources (*P* > 0.2).

**Table 2:** Kinetic parameters of total, essential, and collagen specific, amino acid concentration in plasma after the consumption of collagen and dairy protein sources.

		**Cas**	**HCas**	**Gel**	**Pep**	**LCol**	**BBr**	**Significance**
TAA	Baseline (μmol/L)	2, 156 ± 234	2, 233 ± 403	2, 155 ± 308	2, 178 ± 331	2, 151 ± 325	2, 254 ± 392	*P* = 0.6430
	C_max_ Obs (μmol/L)	3, 012 ± 407	3, 725 ± 641	3, 525 ± 631	3, 710 ± 670	3, 445 ± 641	3, 552 ± 561	*P* = 0.0004
	T_max_ (min) (min/max)	45 ± 35	35 ± 9	59 ± 28	48 ± 27	47 ± 39	64 ± 24	*P* = 0.0877
EAA	Baseline (μmol/L)	998 ± 116	1, 044 ± 170	1, 030 ± 135	1, 017 ± 148	1, 000 ± 147	1, 074 ± 142	*P* = 0.4621
	C_max_ Obs (μmol/L)	1, 457 ± 137	1, 946 ± 292	1, 311 ± 211	1, 313 ± 190	1, 270 ± 198	1, 347 ± 138	*P* <0.0001
	T_max_ min (min/max)	47 ± 36	33 ± 10	47 ± 24	35 ± 31	36 ± 42	51 ± 24	*P* = 0.3310
Proline	Baseline (μmol/L)	168 ± 47	177 ± 49	163 ± 55	173 ± 62	166 ± 49	170 ± 72	*P* = 0.7901
	C_max_ Obs (μmol/L)	282 ± 68	392 ± 71	381 ± 76	431 ± 113	388 ± 89	386 ± 85	*P* <0.0001
	T_max_ min (min/max)	56 ± 34	41 ± 9	61 ± 22	55 ± 27	52 ± 38	103 ± 29	*P* <0.0001
Glycine	Baseline (μmol/L)	229 ± 67	235 ± 64	226 ± 172	215 ± 176	222 ± 143	229 ± 170	*P* = 0.5773
	C_max_ Obs (μmol/L)	267 ± 67	253 ± 64	674 ± 172	747 ± 176	646 ± 143	666 ± 170	*P* <0.0001
	T_max_ min (min/max)	67 ± 49	39 ± 49	71 ± 25	48 ± 20	53 ± 26	88 ± 24	*P* = 0.0103
Lysine	Baseline (μmol/L)	174 ± 33	178 ± 31	179 ± 28	178 ± 58	176 ± 33	183 ± 30	*P* = 0.8818
	C_max_ Obs (μmol/L)	273 ± 31	369 ± 65	269 ± 52	271 ± 50	262 ± 48	272 ± 41	*P* <0.0001
	T_max_ min (min/max)	51 ± 36	29 ± 10	45 ± 19	41 ± 18	44 ± 33	51 ± 17	*P* = 0.1629
Leucine	Baseline (μmol/L)	136 ± 17	144 ± 27	145 ± 26	144 ± 28	140 ± 29	158 ± 29	*P* = 0.1640
	C_max_ Obs (μmol/L)	230 ± 31	351 ± 59	184 ± 29	190 ± 34	181 ± 30	203 ± 28	*P* <0.0001
	T_max_ min (min/max)	47 ± 36	29 ± 10	36 ± 23	29 ± 31	33 ± 41	33 ± 12	*P* = 0.3768

*Data presented as mean ± SD*.

In terms of TAA, C_max_ Obs was similar for all protein sources, except for Cas which was significantly lower (*P* < 0.04) than all protein sources and LCol which was significantly lower than Pep (*P* = 0.03). HCas was significantly higher for C_max_ Obs for EAA than all other protein sources (*P* < 0.0001) while Cas was only higher than BBr and LCol (*P* < 0.04). There was no difference in C_max_ Obs of EAA for any of the collagen protein sources (*P* > 0.1). The C_max_ Obs of proline was similar (*P* > 0.1) for all protein sources other than Cas, which was significantly lower than the others (*P* < 0.001). HCas provided the highest C_max_ Obs for lysine compared to all other protein sources (*P* < 0.002). Collagen proteins provided a higher C_max_ Obs for glycine compared to Cas and HCas (*P* < 0.0001) while there was no difference between the dairy protein sources (*P* = 0.8). Meanwhile, HCas provided the highest C_max_ Obs of leucine compared to all collagen proteins (*P* = 0.0001) and Cas also had a higher C_max_ Obs for leucine than these products (*P* < 0.04). However, differences in C_max_ Obs for leucine between any of the collagen proteins failed to reach significance (*P* > 0.06).

#### Timing of Maximum Concentration (T_max_)

Results of T_max_ in Table 2 illustrate a similar time course for TAA for all products except an earlier peak with HCas than BBr and Gel (*P* < 0.02). There was no difference in T_max_ of EAA with all protein sources peaking between ~ 30 and 50 min (*P* > 0.1). T_max_ of proline for BBr was slower than all other protein sources (*P* < 0.002). Although the time to peak for proline was slower for the non-hydrolysed proteins compared to the hydrolysed proteins, this did not reach statistical significance (*P* > 0.05). BBr was the slowest to peak for glycine, and was significantly slower than Pep and LCol (*P* < 0.001). The T_max_ of glycine for Gel was also found to be slower than for Pep (*P* = 0.04). There was no difference in T_max_ for leucine between any of the protein sources, with all peaking between ~ 30 to 50 min (*P* > 0.04). The only difference for lysine T_max_ was a quicker peak with BBr than HCas (*P* = 0.001).

#### Area Under the Curve (AUC)

**Figure 2:**
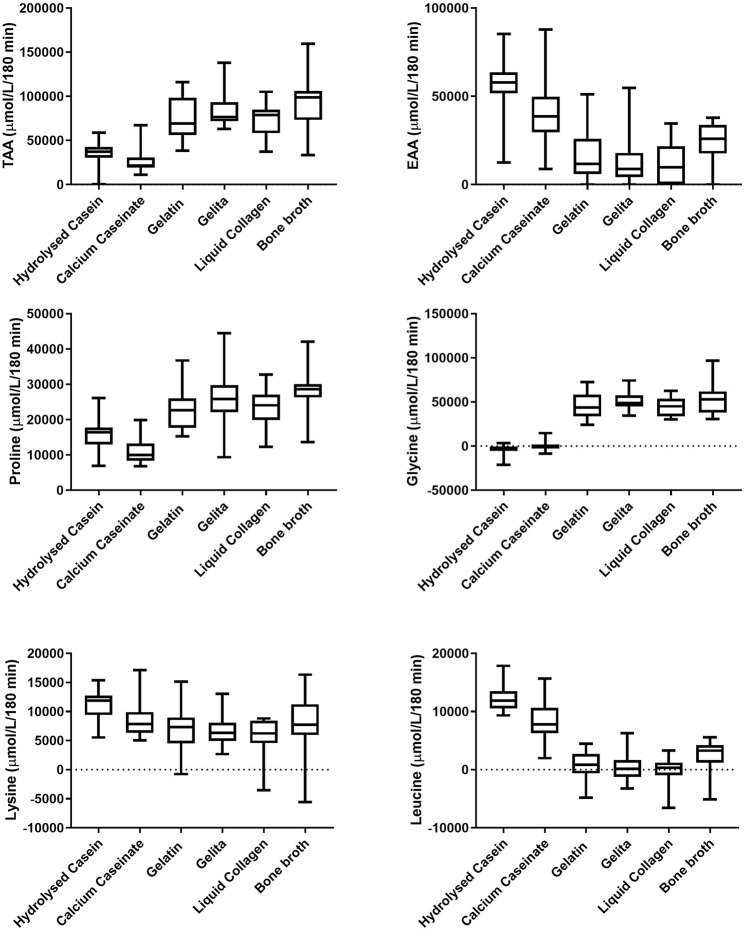
Area under the concentration—time curve (AUC) of plasma amino acids over 180 min after the consumption of dairy and collagen protein sources. Middle line shows mean, boxes represent 25–75th percentile and whiskers represent min and max values.

Figure 2 displays the AUC of amino acid concentrations in plasma after consumption of the protein sources. BBr provided the highest AUC for TAA at 91,029 ± 29,630 μmol/L/180 min, which was significantly higher than both dairy proteins [Cas and HCas at 27,265 ± 14,130 μmol/L/180 min and 36,042 ± 12,833 μmol/L/180 min, respectively (*P* < 0.0001)] but not significantly different to any of the collagen protein supplements [7,7052 ± 2,4304, 84,664 ± 20,582 and 71,882 ± 19,085 μmol/L/180 min for Gel, Pep and LCol, respectively (*P* > 0.2)]. However, as seen in Figure 1, plasma AAs after consumption of BBr remained elevated at the completion of 180 min, particularly for proline and glycine. Cas provided the lowest AUC for TAA, and was significantly lower than all collagen supplements (*P* < 0.0002), but not HCas (*P* = 0.6). Although hydrolysed supplements appeared to have a higher AUC for TAA than their non-hydrolysed counterparts, this was not statistically different for either collagen or dairy proteins (*P* > 0.6). Collagen proteins had a higher AUC than dairy proteins for TAA (81,157 ± 8,419 and 31,654 ± 6,206 μmol/L/180 min, respectively, *P* < 0.0001).

HCas provided the highest AUC for EAA at 55,992 ± 15,675 μmol/L/180 min, which was significantly greater than all other protein sources (*P* < 0.003), except Cas which provided an AUC for EAA of 39,587 ± 18,573 μmol/L (*P* = 0.2). The AUC for EAA of Cas, was only significantly higher than LCol and Pep which provided 8,806 ± 16,410 and 12,238 ± 13,682 μmol/L/180 min, respectively (*P* < 0.006). The lowest AUC of EAA was provided by LCol at 8,806 ± 16,410 μmol/L/180 min, but this was not different to the other collagen protein sources (*P* > 0.8).

In terms of individual AA, the AUC of lysine was greater for HCas than Gel, Pep and LCol [AUC of 11,187 ± 2,764 μmol/L/180 min compared to 7,003 ± 4,340, 6,310 ± 2,714, and 5,729 ± 3,290 μmol/L/180 min, respectively (*P* < 0.008)]. For leucine, dairy proteins had a higher AUC than all collagen proteins at 10,187 ± 3,023 μmol/L/180 min and 746 ±1,043 μmol/L/ 180 min, respectively (*P* < 0.001). Meanwhile, HCas provided a higher AUC for leucine than Cas (*P* = 0.003). Collagen proteins has a higher AUC than dairy proteins for proline (25,102 ± 2,345 and 13,547 ± 3,450 μmol/L/180 min, for Gel, Pep, and LCol, respectively *P* < 0.01), and glycine [(48,563 ± 4,429 and −1,863 ± 2,255 μmol/L/ 180 min (*P* < 0.0001)].

#### Collagen and Dairy Protein Acceptability and Palatability

Responses to the questions around acceptability and palatability for each protein source are displayed in Table 3. LCol had the highest scores individually, and on average, while HCas was rated the lowest. Despite these differences between products, only 53% of participants reported being willing to pay the specified price for either product. Meanwhile, the non-hydrolysed supplements (which were also the lowest in cost; Table 1), scored 93% for willingness to pay, if it was found to be beneficial to connective tissues.

**Table 3:** Likert scale of protein source feedback and palatability.

**Question (1–7)**	**Cas**	**HCas**	**Gel**	**Pep**	**LCol**	**BBr**	**Significance**
Serve size	20 g	20 g	20 g	20 g	60 mL	300 mL	*P* <0.001
1	4 (1–7)	3 (1–5)	4 (1–7)	5 (2–7)	6 (307)	5 (2–7)	*P* <0.001
2	6 (3–7)	5 (2–6)	6 (2–7)	6 (2–7)	7 (4–7)	6 (2–7)	*P* = 0.032
3	6 (2–7)	5 (1–7)	6 (2–7)	6 (2–7)	7 (5–7)	6 (2–7)	*P* = 0.044
4	6 (3–7)	4 (1–7)	6 (1–7)	6 (2–70)	7 (4–7)	6 (2–7)	*P* = 0.006
5 (% Yes)	93	53	93	60	53	73	-
^*^Question 1: The palatability (taste, texture) of the product was acceptable.							
^*^Question 2: The volume of the product (300 mL) was acceptable to consume in the time specified (5 min).							
^*^Question 3: The volume of the product would be acceptable to consume within 20–40 min prior to exercise.							
^*^Question 4: I would be willing to consume this product at least twice a day as part of a rehabilitation program.							
^**^Question 5: Would you be willing to pay the specified amount for one serve of the product if it was beneficial to connective tissue health?							

*Data presented as median (range)*.

*1, strongly agree; 7, strongly disagree*.

* *Out of 1 (strongly agree) to 7 (strongly disagree)*.

** *% out of 100*.

## Discussion

A number of AAs have been suggested to play a complementary role in the synthesis of collagen in ligaments and tendons, when combined with an appropriate mechanical stimulus (39). There is emerging evidence that the ingestion of key AAs (including proline, glycine, lysine, and leucine), or combinations thereof, provide enhanced availability to support the synthesis of collagen when combined with an appropriate exercise protocol (42). Accordingly, the aim of the present study was to characterize the bioavailability (e.g., timing of appearance, maximum concentration, and AUC) of TAA, EAA, and key AAs that may be involved in the synthesis of collagen, after the ingestion of a selection of collagen and dairy protein sources.

In general, plasma AA responses reflected the AA profiles of the consumed supplement, except in the case of the casein, and bone broth. These both resulted in a lower and more prolonged appearance of AAs in the blood over the 180 min following consumption (See Tables 1, 2 and Figure 2). This is likely due to various components known to slow gastric emptying such as the fat content of the BBr (43), and the clotting of casein in the stomach after ingestion (44). However, it is also plausible that the delayed gastric emptying of BBr may be related to the volume was consumed. As can be seen in Figure 1, the plasma concentration of AAs after consumption of BBr remained elevated at the 180 min mark, and most likely would have continued to be available at a higher amounts in the blood beyond the 3 h that were monitored for this study.

It has been proposed that AAs play a supportive, rather than stimulatory role in the synthesis of collagen within ligaments and tendons (21, 39, 45). Meanwhile, exercise has been shown to be a potent regulator of collagen turnover resulting in an upregulation of collagen synthesis for a period of up to 72 h (46). Exercise results in an increase in hormones that have been shown to stimulate the synthesis of collagen in connective tissue (e.g., growth hormone, and insulin-like growth factor 1) (47). As ligaments and tendons are poorly vascularised tissues, it may be sensible to isolate the provision of AAs to scenarios involving the exercise-induced enhancement of blood flow (48). Therefore, protein sources that achieve higher AA peaks over a shorter period of time (e.g., HCas) may be considered optimal, whereas a slower release of key AAs over an extended period of time (e.g., with BBr) may not be as easily matched to enhanced tissue blood flow. Literature to date suggests that there is dose related response to the key AA involved in the synthesis of collagen. Indeed, Shaw et al. (21) illustrated that a 15 g dose of gelatin resulted in an increased availability of AAs and collagen synthesis than a 5 g dose, resulting in improvements to tissue mechanics in engineered ligaments *in vitro*. Peak blood concentrations of glycine and proline in the current study (~650–750 and 350–450 μmol/L, respectively) were slightly higher than those reported in previous work [i.e., 448 ± 165 and 238 ± 77 μmol/L in Shaw et al. (21)]. This is to be expected given the slightly higher dose within our current protocol. However, others have shown similar plasma values after the ingestion of 35 g of collagenous protein (32) which they suggest shows an upper threshold to the AA availability of collagen proteins. In general since all AAs appeared to peak between 30 and 60 min (Table 2), the consumption of ~ 20 g of protein within the 30–60 min prior to exercise would ensure the optimal availability of AAs at a time when synthetic machinery is upregulated, and tissue perfusion supported (13, 46, 49). It should be noted, however, that whether the increased availability of AAs within the plasma results in an increased availability of AAs around the target tissue (e.g., the peritendinous fluid), where it is able to be utilized and integrated into the tissue is yet to be determined.

While the maximum concentrations of total AA present within the plasma after the ingestion of collagen and high leucine dairy proteins were comparable, dairy proteins provided a larger C_max_ Obs and AUC of leucine, while hydrolysed casein provided a higher C_max_ Obs of EAA than both dairy and collagen proteins, and collagen proteins were superior in terms of glycine. Such differentiation mean that no protein source was superior in terms of all AAs that are potentially implicated in collagen synthesis. If subsequent research identifies the benefits of EAA as well as collagen precursors (e.g., proline and glycine), a case could be made for the consistent intake of EAA (i.e., the current guidelines for regular intake of high-quality protein sources over the day), with a supplemental and/or food source of collagenous protein consumed at key periods i.e., prior to exercise. The lack of such a pattern may also detract from the health of connective tissues.

It has been proposed that hydrolysed proteins are superior to non-hydrolysed forms in providing a higher AA bioavailability; more specifically, being able to reach a target tissue more quickly and with a higher peak concentration (31, 32, 50). Within the present study, the plasma characteristics of hydrolysed and non-hydrolysed dairy proteins showed some differences, but these were not seen with the collagen proteins. Our findings are similar to the study of Koopman and co-workers where ingestion of 35 g of hydrolysed casein protein resulted in an increased bioavailability and incorporation rate of AAs into skeletal muscle protein than intact casein (50). However, while another more recent study showed that the ingestion of 35 g of enzymatically hydrolysed collagen resulted in a higher bioavailability of several AAs compared to non-enzymatically hydrolysed collagen (32), we did not find any difference in the bioavailability of gelatin compared to a hydrolysed peptide powder. It is possible that differences in processing methods accounted for this finding as it has been suggested to influence digestibility and therefore bioavailability of AAs (51).

In terms of the limitations of this study, we acknowledge that the questionnaire relating to acceptance and palatability of the protein sources used in this study was not a validated tool. Nevertheless, it provided insight into factors that are likely to affect the compliance of the use of protein sources that have potential benefit for collagenous tissues. Indeed, it indicated that despite the superiority of HCas over Cas in terms of AA availability, its poor scores for taste/texture are likely to affect consumer uptake and compliance with regular use. On the other hand, non-hydrolysed supplements scored well in terms of acceptability in taste and cost; this makes gelatin a potentially useful product since it provided similar peak and total AA exposure than other collagen products. Furthermore, our inability to measure the composition of the protein sources actually used in this study until after its conduct meant that we relied on previously collected data, or that stated by the manufacturer which differed to the final measured content (Table 1). The subsequent discovery of the higher protein of this broth makes it difficult to compare the bioavailability based on their actual protein intake. However, since the serves used in this study are the commonly consumed or recommended amounts of these protein sources, it did allow real-life insight into the AA profiles.

In summary, our study characterized the blood AA profiles following the intake of a range of dairy and collagen supplements providing 20 g of protein, and a common serve of beef broth of higher total protein. While protein sources of similar origin contained a comparable total AA profile, consumption of dairy proteins provided a more pronounced amount of EAA and leucine than collagen sources, with hydrolysed casein showing increased bioavailability of AAs than an intact form. Intake of collagen proteins achieved a greater peak concentration of glycine, with small differences between hydrolysed and non-hydrolysed forms. Although the total AA content of the bone broth was greater, a lower and more sustained blood AA profile seems likely. This information may help to inform protocols for achieving ideal blood AA responses once the optimal support for the synthesis of collagenous tissues is identified. However, the cost and palatability of these dietary and supplement sources should be considered.

## Data Availability Statement

All datasets generated for this study are included in the manuscript/Supplementary Files.

## Ethics Statement

The studies involving human participants were reviewed and approved by The Human Ethics Committee of the Australian Institute of Sport. The patients/participants provided their written informed consent to participate in this study.

## Author Contributions

RA, LB, and GS were involved in study design. RA and NT was involved in data collection, data analysis, and statistical analysis RA, LB, GS, and NT were involved in results interpretation, drafting, reviewing, and revising the initial manuscript.

### Conflict of Interest

The authors declare that the research was conducted in the absence of any commercial or financial relationships that could be construed as a potential conflict of interest.
