# Supplementary material for: Plasma Amino Acid Concentrations After the Ingestion of Dairy and Collagen Proteins, in Healthy Active Males
Source: Front Nutr. 2019 Oct 15;6:163. doi: 10.3389/fnut.2019.00163 (PMC6803522; doi:10.3389/fnut.2019.00163)
Supplement: Supplementary file 1 [file Table_1.docx]

**Supplementary Table 1. Amino acid profiles of select food/supplements (Mg/serve)**

|  | Cas | HCas | Gel | Pep | LCol | BBr |
| --- | --- | --- | --- | --- | --- | --- |
| Hyp | N.D. | N.D. | 2330 | 2434 | 2052 | 5910 |
| Gly | 360 | 354 | 4750 | 4904 | 4212 | 11760 |
| Pro | 2038 | 2060 | 2664 | 2722 | 2406 | 6420 |
| Hyl | N.D. | N.D. | 210 | 208 | 186 | 570 |
| Lys | 1422 | 1468 | 696 | 746 | 630 | 1740 |
| Leu | 1782 | 1786 | 588 | 584 | 516 | 1710 |
| His | 546 | 480 | 140 | 158 | 132 | 450 |
| Tau | N.D. | N.D. | N.D. | N.D. | N.D. | N.D. |
| Ser | 1086 | 1088 | 668 | 702 | 582 | 1680 |
| Arg | 706 | 700 | 1584 | 1652 | 1380 | 3840 |
| Asp | 1212 | 1238 | 1072 | 1088 | 1996 | 2970 |
| Glu | 3930 | 4040 | 1956 | 1970 | 1788 | 5670 |
| Thr | 810 | 812 | 356 | 352 | 306 | 990 |
| Ala | 540 | 540 | 1694 | 1678 | 1530 | 4560 |
| Tyr | 994 | 990 | 102 | 114 | 42 | 360 |
| Met | 550 | 554 | 160 | 154 | 138 | 420 |
| Val | 1234 | 1242 | 450 | 474 | 396 | 1260 |
| Iso | 986 | 986 | 300 | 252 | 264 | 810 |
| Phe | 976 | 978 | 388 | 402 | 336 | 1140 |
| Total protein (g) | 19.2 | 19.4 | 20 | 20 | 17.9 | 52.2 |
| HYP: Hydroxyproline; GLY: Glycine; Pro: Proline; HYL: Hydroxylysine; Lys: Lysine; Leu: Leucine; HIS: Histidine; TAU: Taurine; SER: Serine; ARG: Arginine; GLU: Glutamic acid; THR: Threonine; ALA: Alanine; TYR: Tyrosine; MET: Methionine; VAL: Valine; ISO: Isoleucine; PHE: Phenylalanine; N.D.: Not detected. | | | | | | |
